# New insight into the analgesic recipe: A cohort study based on smart patient-controlled analgesia pumps records

**DOI:** 10.3389/fphar.2022.988070

**Published:** 2022-10-10

**Authors:** Yiyan Song, Qiulan He, Wenzhong Huang, Lu Yang, Shaopeng Zhou, Xiaoyu Xiao, Zhongxing Wang, Wenqi Huang

**Affiliations:** ^1^ Department of Anesthesia, The First Affiliated Hospital of Sun Yat-sen University, Guangzhou, China; ^2^ Department of Epidemiology and Preventive Medicine, School of Public Health and Preventive Medicine, Monash University, Melbourne, VIC, Australia; ^3^ Department of Anesthesia, The Fifth Affiliated Hospital of Sun Yat-sen University, Zhuhai, China

**Keywords:** patient-controlled analgesia, sufentanil, hydromorphone, flurbiprofen, postoperative nausea and vomiting, generalized estimation equation, analgesic insufficiency, dizziness

## Abstract

**Purpose:** Intravenous patient-controlled analgesia (IV-PCA) has been widely used; however, regimen criteria have not yet been established. In China, the most often used opioid is sufentanil, for which repeated doses are a concern, and empirical flurbiprofen axetil (FBP) as an adjuvant. We hypothesized that hydromorphone would be a better choice and also evaluated the effectiveness of FBP as an adjuvant.

**Methods:** This historical cohort study was conducted in two tertiary hospitals in China and included 12,674 patients using hydromorphone or sufentanil for IV-PCA between April 1, 2017, and January 30, 2021. The primary outcome was analgesic insufficiency at static (AIS). The secondary outcomes included analgesic insufficiency with movement (AIM) and common opioid-related adverse effects such as postoperative nausea and vomiting (PONV) and dizziness.

**Results:** Sufentanil, but not the sufentanil-FBP combination, was associated with higher risks of AIS and AIM compared to those for hydromorphone (OR 1.64 [1.23, 2.19], *p* < 0.001 and OR 1.42 [1.16, 1.73], *p* < 0.001). Hydromorphone combined with FBP also decreased the risk of both AIS and AIM compared to those for pure hydromorphone (OR 0.74 [0.61, 0.90], *p* = 0.003 and OR 0.80 [0.71, 0.91], *p* < 0.001). However, the risk of PONV was higher in patients aged ≤35 years using FBP (hydromorphone-FBP vs. hydromorphone and sufentanil-FBP vs. hydromorphone, OR 1.69 [1.22, 2.33], *p* = 0.001 and 1.79 [1.12, 2.86], *p* = 0.015).

**Conclusion:** Hydromorphone was superior to sufentanil for IV-PCA in postoperative analgesia. Adding FBP may improve the analgesic effects of both hydromorphone and sufentanil but was associated with an increased risk of PONV in patients <35 years of age.

## Introduction

Proper management of postoperative pain is important as it may reduce the length of hospital stay and the incidence of complications including atelectasis, pneumonia, and thromboembolism (Muehling et al., 2008; Tenenbein et al., 2008; Ghosh and Chatterji, 2019; Turan et al., 2019). Patient-controlled analgesia (PCA) is a widely used technique that allows personalized dosing and timely access to pain medication. Intravenous PCA (IV-PCA) is one of the most favored modalities due to its convenience (Momeni et al., 2006). Unexpectedly, PCA showed small advantages over conventional non-patient-controlled analgesia in achieving lower pain scores, as supported by moderate to low-level evidence, with higher opioid consumption (Hudcova et al., 2006; McNicol et al., 2015). Although opioids remain the main analgesics of IV-PCA, their effectiveness in clinical practice is restricted by their side effects (Momeni et al., 2006), mainly postoperative nausea and vomiting (PONV), respiratory depression, dizziness, etc. Adjuvants including NSAIDs, lidocaine, clonidine, dexmedetomidine, and magnesium have been evaluated for their effectiveness in improving analgesic efficiency and reducing opioid-related side effects by reducing opioid consumption, (Yy et al., 1998; Burstal et al., 2001; Jeffs et al., 2002; Unlügenç et al., 2003; Nie et al., 2018; Xiang et al., 2018; Shim and Gan, 2019). However, these studies rarely found differences in side effect profiles between different opioids.

According to a recent national survey in Chinese hospitals, sufentanil ranked first in opioids used for PCA (>80% of hospitals) (Wang et al., 2021). Due to the lack of a well-established standard, the choice of opioid and adjuvant in IV-PCA depends on anesthesiologist familiarity and drug accessibility, rather than evidence (Grass, 2005; Fernandes et al., 2017). Although sufentanil has been proven to be effective in IV-PCA, its extremely rapid onset and short duration have raised concerns about repeated dosing (Lehmann et al., 1991; Minkowitz et al., 2013). Moreover, despite its higher plasma protein binding (∼90% vs. 8%–19%), the free fraction of sufentanil was more dependent on total drug concentration and volume balance, while the free fraction of hydromorphone was nearly constant (Saari et al., 2014; Drugs.com, 2021a; Drugs.com, 2021b). However, few studies have compared analgesic efficacy and adverse effects between hydromorphone and sufentanil in postoperative IV-PCA, with conflicting results (Yan et al., 2018; Yang et al., 2018). In addition to opioids, flurbiprofen axetil (FBP), a nonsteroid anti-inflammatory drug (NSAID) that is commonly administered on a scheduled rather than on an as-needed basis, is often empirically used as an adjuvant for IV-PCA in Chinese hospitals (Wick et al., 2017; Shi et al., 2021). However, gastrointestinal adverse effects occur more frequently with FBP than with other NSAIDs, among which nausea is representative with an incidence of >3% (Brogden et al., 1979; Drugs.com, 2022). Therefore, we hypothesized that hydromorphone might be a better choice and performed further evaluations of the effectiveness of FBP (Wang et al., 2021).

This study compared the efficacy and adverse effect profiles between hydromorphone and sufentanil and evaluated the effect of adding FBP as an adjuvant to the IV-PCA pump. With the help of intelligent PCA pumps, this study included a larger population with higher coverage of the postoperative period than traditional PCA research to provide evidence to inform IV-PCA formulation.

## Methods

### Study design

We conducted this retrospective cohort study in two tertiary medical centers in Guangdong, China. The study included patients who underwent surgery in one of five specialties (gynecology [GYN], major abdominal, thoracic, orthopedics, or urology) between April 1, 2017, and January 30, 2021. The requirement for written consent was waived and this study was approved by the ethics committees of the First and Fifth Affiliated Hospitals of Sun Yat-sen University according to the China Good Clinical Practice (GCP) guidelines and the tenets of the Declaration of Helsinki.

### Data source

The data used in this study were extracted from the analgesic information database of the RHEN^®^ PCA infusion pump system (RHEN Meditech Inc., Jiangsu, China), which recorded infusion activities and synchronized patient characteristics and surgery information from the DoCare^®^ anesthesia clinical information system (MedicalSystem Co., Ltd., Suzhou, China). Infusion data such as the number of patient bolus attempts, the number of valid boluses, and the total volume delivered in milliliters was automatically documented every 20 min by the RHEN^®^ smart PCA pump. The analgesic recipes and postoperative assessments were recorded manually by anesthesiologists. Multifaceted evaluation of analgesic effects using the visual analogue scale (VAS, range 0–10) to measure pain intensity both at static and with movement, and opioid-related side effects such as PONV, dizziness, sedation, confusion, respiratory depression, and decreased muscle strength was performed by the acute pain service (APS) team that regularly visited the wards. The end time of the surgery varied by individual; hence, the analgesic evaluations covered most of the postoperative time points.

### Study patients

**FIGURE 1:**
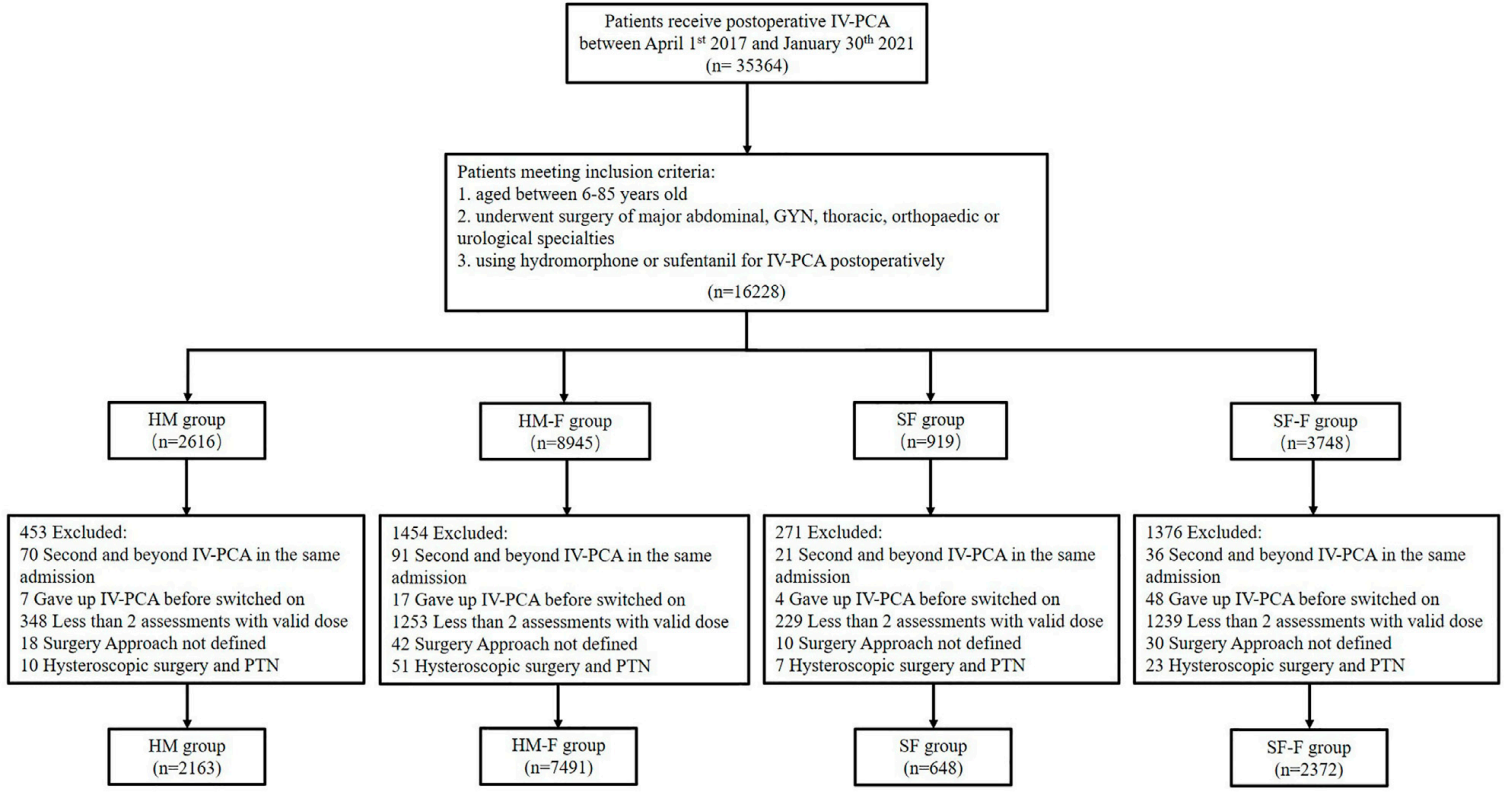
Flow chart showing the study organization, including patient inclusion, exclusion, and classification.

This study included patients aged 6–85 years who underwent one of the five surgical specialties described above and received either hydromorphone or sufentanil for IV-PCA during the study period. The exclusion criteria included: 1) second and beyond PCA therapy in the same admission; 2) discontinued IV-PCA use for personal reasons or mechanical failure before the pump was switched on; 3) <2 postoperative analgesic assessments with valid real-time doses; 4. unknown surgery approaches for GYN, major abdominal, and urologic surgery; 5) hysteroscopic surgery and percutaneous nephrolithotripsy (PTN) (due to extremely small sample sizes). To improve the model efficiency, assessments beyond the sixth record for each participant were also removed (*n* = 99, mostly >48 h after surgery). Patients were assigned to the hydromorphone (HM) or sufentanil (SF) groups if they received hydromorphone or sufentanil, respectively, as single opioids for IV-PCA without adding FBP as an adjuvant. In contrast, patients receiving FBP were assigned to the hydromorphone-flurbiprofen (HM-F) or sufentanil-flurbiprofen (SF-F) groups according to the opioid used. The workflow is shown in Figure 1.

### Institutional standards for anesthesia and analgesic practice

In the two institutions included in this study, the anesthesiologists were randomly assigned to surgeries. General anesthesia was induced by target-controlled infusion of propofol (3–6 g/ml) and injection of sufentanil (0.3–0.5 g/kg) and muscle relaxants (rocuronium of cis-atracurium). Anesthesia was routinely maintained with target-controlled infusions of propofol (2–3 g/ml) and remifentanil (2–4 ng/ml) in routine, while sevoflurane (1%–2%) was added according to patient status and anesthesiologist preference. Additional intraoperative sufentanil was administered at the discretion of the anesthesiologist. NSAIDs and serotonin receptor-3 antagonists were routinely administered 1h before the end of surgery. Upon surgery completion, IV-PCA was started, and the patients were transferred to the postanesthetic care unit, where they received extubation and extra analgesia as needed. The choice of opioid in IV-PCA was hydromorphone (50–80 g/ml), sufentanil (0.6–1 g/ml) or other opioids, as decided solely by the anesthesiologist.

The parameters for the intravenous analgesia pump (RHEN Meditech Inc., Nantong, Jiangsu province, ISO9001:2008) were as follows: background dose 0–2 ml/h, PCA dose 2–3 ml, lockout time 10–15 min, and hour limit 10–16 ml. After analgesic assessment, the parameter settings were adjusted according to the following rules: 1) the background dose was increased by 25% if the VAS was static at >3; 2) the PCA dose was increased by 25% and the lockout time was reduced by 25% if the VAS with movement was >5 despite appropriate patient pump control or the hourly limit was reached. A rescue dose (usually 0.05–0.1 g tramadol) was prescribed by the surgeons according to patient complaints.

### Outcomes

The primary outcome was analgesic insufficiency at static (AIS, defined as VAS at static >3). The secondary outcomes were analgesic insufficiency with movement (AIM, defined as VAS with movement >3), PONV, and dizziness. The VAS cutoff was chosen because the patients were instructed to maintain a pain score of 1–3. This study did not consider other opioid relative adverse effects such as sedation, confusion, respiratory depression, and decreased muscle strength because their extremely low incidences made it difficult to draw reliable conclusions.

### Covariates

Among all patient characteristics, our study included only age and sex. Height and weight were not considered as previous studies demonstrated no correlation between patient weight and morphine consumption (Grass, 2005). Surgical type was classified according to specialties and approaches, with 11 levels (laparoscopic GYN, open GYN, laparoscopic abdominal, open abdominal, limb, spine, open thoracic, video-assisted thoracoscopy surgery [VATS], laparoscopic urologic, cystoscopy and ureteroscopy, and open urologic). Time was a continuous covariate representing the duration since the end of the operation, which was also the start of IV-PCA pump use. The infused doses of hydromorphone (m_H_), sufentanil (m_S_), and flurbiprofen (m_F_) were defined as the doses of these drugs that were infused at the time of assessment. Opioid consumption was calculated as morphine milligram equivalent using the multipliers 4 or 0.5 for hydromorphone and sufentanil, respectively.

### Statistical analysis

Baseline characteristics were calculated and stratified by group. Normally distributed variables were shown as means±standard deviations. Analysis of variance (ANOVA) and Tukey HSD tests were used for intergroup comparisons. Categorical variables were shown as cases and frequencies and compared using chi-square tests. A two-level (individual and hospital) generalized estimation equation (GEE) model with an exchangeable correlation matrix was used to account for the clustering effect introduced by repeated measurements of an individual and the heterogeneous populations of different hospitals. The covariates in the GEE model included sex, age, surgery type, and time. Infused doses, including such as m_H_, m_S_, and m_F_, were also controlled in the model for comparisons between groups. Spline functions were used to address nonlinearity between the risk of developing analgesic insufficiency and time. The optimal parameters were chosen to minimize the quasi-likelihood under the independence model information criterion (QIC). The HM group was chosen as the reference in multigroup comparisons.

As this study included pediatrics, adults, and geriatrics and age is an important factor affecting analgesic selection, a stratified analysis according to age was further performed to assess the association between treatment group and outcomes that might vary with age (≤35 years, 35–65 years, or ≥65 years), sex and surgery type. The estimated incidence of the outcomes over time was calculated using an explorative GEE model. Differences with *p* < 0.05 were considered significant. All analyses were conducted using R software (version 4.0.5).

### Sample size

Due to the retrospective cohort study design, the sample size was based on available data. No formal statistical power calculation was conducted.

### Missing data

Only complete data were used in the analyses.

## Results

### Patients

Finally, a total of 12,674 patients and 34,926 observations were identified from the PCA pump database (Figure 1). Most of the study population received a hydromorphone-based recipe, among which 7,491 (59.1%) received FBP as adjuvant and 2,163 (17.1%) did not. Sufentanil-based recipes were administered less frequently, with 648 (5.1%) and 2,372 (18.7%) in the SF and SF-F groups, respectively. The sufentanil-based groups showed significantly higher average opioid consumption than those in the hydromorphone-based groups (HM: 7.07 [5.59], HM-F: 7.42 [4.37], SF: 15.94 [11.08], SF-F: 17.56 [10.91], *p* < 0.001]. The average opioid consumption was significantly higher in the SF-F group compared to that in the SF group (*p* < 0.001), but similar between the HM and HM-F groups (*p* = 0.138). These four groups differed significantly in baseline characteristics including age, sex, surgery type, and distribution of analgesic assessments, as summarized in Table 1.

**TABLE 1:** Characteristics of the study cohort.

Characteristic	HM (n = 2163)	HM-F (n = 7491)	SF (n = 648)	SF-F (n = 2372)	P value^a^
Age, mean(SD), y	50.29 (21.31)	51.46 (15.29)	54.49 (19.32)	51.38 (14.58)	<0.001
Sex, No.(%)	—	—	—	—	<0.001
Male	1154 (53.4)	3464 (46.2)	315 (48.6)	962 (40.6)	
Female	1009 (46.6)	4027 (53.8)	333 (51.4)	1410 (59.4)	
Height, mean (SD), cm	161.26 (10.69)	162.91 (8.02)	161.67 (9.79)	162.49 (7.82)	<0.001
Weight, mean (SD), kg	58.60 (13.13)	60.09 (10.98)	59.06 (12.13)	59.80 (10.73)	<0.001
Surgery type, No.(%)	—	—	—	<0.001	—
Laparoscopic GYN	72 (3.3)	659 (8.8)	24 (3.7)	213 (9.0)	—
Open GYN	138 (6.4)	885 (11.8)	46 (7.1)	345 (14.5)	—
Laparoscopic abdominal	228 (10.5)	950 (12.7)	94 (14.5)	333 (14.0)	—
Open abdominal	506 (23.4)	1961 (26.2)	125 (19.3)	570 (24.0)	—
Limb surgery	287 (13.3)	582 (7.8)	84 (13.0)	146 (6.2)	—
Spine surgery	217 (10.0)	510 (6.8)	50 (7.7)	152 (6.4)	—
Open thoracic	196 (9.1)	805 (10.7)	74 (11.4)	239 (10.1)	—
VATS	83 (3.8)	270 (3.6)	36 (5.6)	168 (7.1)	—
Laparoscopic urologic	84 (3.9)	169 (2.3)	31 (4.8)	39 (1.6)	—
Cystoscopy and ureteroscopy	42 (1.9)	92 (1.2)	10 (1.5)	26 (1.1)	—
Open urologic	310 (14.3)	608 (8.1)	74 (11.4)	141 (5.9)	—
Average opioid consumption mean (SD), mg/d	7.07 (5.59)	7.42 (4.37)	15.94 (11.08)	17.56 (10.91)	<0.001
Observations per patient, No. (%)	—	—	—	—	<0.001
2	642 (29.7)	2087 (27.9)	208 (32.1)	802 (33.8)	—
3	1426 (65.9)	5124 (68.4)	405 (62.5)	1461 (61.6)	—
≥4	95 (4.4)	280 (3.7)	35 (5.4)	109 (4.6)	—
Time of first assessment, median (IQR), h	9.86 (6.76, 14.62)	9.64 (6.18, 14.26)	10.69 (7.32, 15.89)	11.61 (7.61, 19.04)	<0.001

Abbreviations: HM, Hydromorphone; HM-F, Hydromorhone-Flubiprofen Axetil; SF, Sufentanil; SF-F, Sufentanil-Flurbiprofen Axetil; GYN, gynecologic; VATS, Video-assisted thoracoscopic surgery.

^a^ Continuous variables were compared using analysis of variance if they followed normal distribution, otherwise compared with Kruskal-Wallis test, and categorical variables were compared using chi-squared test.

### Primary analysis

Multivariate analysis of the outcomes revealed a higher risk of AIS in the SF group compared to the HM group, which was not observed when FBP was added (SF vs. HM: OR 1.64 [1.23, 2.19], *p* < 0.001 and SF-F vs. HM: OR 1.08 [0.84, 1.38], *p* = 0.561). Moreover, the risk of AIS decreased by 26% in the HM-F group compared to the HM group (OR 0.74 [0.61, 0.90], *p* = 0.003). The differences in AIM between groups were similar to those at static, with the HM-F group showing a lower risk (OR 0.81 [0.71, 0.91], *p* < 0.001).

Both the HM-F and SF-F groups administered FBP as an adjuvant showed higher risks of PONV compared to that in the HM group (OR 1.20 [1.03, 1.40], *p* = 0.018, and OR 1.27 [1.04, 1.55], *p* = 0.021 respectively). The risk of dizziness also increased in the HM-F group compared to that in the HM group (OR 1.28 [1.01, 1.62], *p* = 0.040). PONV and dizziness did not differ between sufentanil and hydromorphone. The results are summarized in Table 2.

**TABLE 2:** Multivariable analysis of factors associated with the primary outcome and secondary outcome.

	Adjusted Odds Ratio (95% CI)			
	AIS	AIM	PONV	Dizziness
Treatment Group	—	—	—	—
HM	1.00 (reference)	1.00 (reference)	1.00 (reference)	1.00 (reference)
HM-F	**0.74 (0.61,0.90)**	**0.80 (0.71,0.91)**	**1.20 (1.03,1.40)**	**1.28 (1.01,1.62)**
SF	**1.64 (1.23,2.19)**	**1.42 (1.16,1.73)**	0.91 (0.70,1.19)	1.14 (0.79,1.63)
SF-F	1.08 (0.84,1.38)	1.10 (0.93,1.30)	**1.27 (1.04,1.55)**	1.25 (0.92,1.69)
Sex	—	—	—	—	
Male	1.00 (reference)	1.00 (reference)	1.00 (reference)	1.00 (reference)	
Female	**1.18 (1.03,1.36)**	**1.27 (1.17,1.39)**	**3.00 (2.69,3.35)**	**1.97 (1.68,2.30)**	
Age^*^	**0.99 (0.99,0.99)**	**0.99 (0.99,0.99)**	**0.99 (0.99,0.99)**	1.00 (0.99,1.00)	
Surgery type	—	—	—	—	
Laparoscopic GYN	1.00 (reference)	1.00 (reference)	1.00 (reference)	1.00 (reference)	
Open GYN	1.23 (0.93,1.63)	**1.33 (1.10,1.61)**	0.87 (0.73,1.03)	0.80 (0.63,1.02)	
Laparoscopic abdominal	0.96 (0.71,1.30)	**1.42 (1.17,1.73)**	0.83 (0.68,1.00)	0.80 (0.60,1.07)	
Open abodominal	**1.45 (1.10,1.90)**	**1.63 (1.36,1.95)**	**0.80 (0.67,0.94)**	**0.73 (0.57,0.93)**	
Limb surgery	0.89 (0.64,1.24)	1.08 (0.87,1.33)	**0.78 (0.64,0.96)**	**0.69 (0.52,0.93)**	
Spine surgery	**0.69 (0.47,0.99)**	1.11 (0.88,1.39)	**0.63 (0.50,0.79)**	**0.39 (0.27,0.57)**	
Open thoracic	**1.79 (1.34,2.39)**	**2.20 (1.81,2.67)**	**0.77 (0.63,0.95)**	0.92 (0.70,1.20)	
VATS	1.25 (0.84,1.85)	**1.87 (1.47,2.39)**	0.81 (0.62,1.07)	0.82 (0.55,1.21)	
Laparoscopic urologic	0.89 (0.53,1.48)	1.08 (0.80,1.46)	1.02 (0.75,1.37)	0.70 (0.43,1.14)	
Cystoscopy and ureteroscopy	0.78 (0.37,1.64)	0.63 (0.39,1.04)	0.80 (0.51,1.26)	**0.25 (0.10,0.61)**	
Open urologic	1.07 (0.78,1.48)	**1.36 (1.10,1.68)**	0.97 (0.79,1.19)	**0.71 (0.53,0.95)**	
m_H_^*^	**1.08 (1.02,1.14)**	**1.13 (1.08,1.18)**	1.00 (0.95,1.04)	**1.10 (1.03,1.17)**	
m_S_^*^	**1.01 (1.00,1.01)**	**1.01 (1.00,1.01)**	1.00 (0.99,1.00)	1.00 (1.00,1.01)	
m_F_^*^	1.00 (1.00,1.00)	1.00 (1.00,1.00)	1.00 (1.00,1.00)	1.00 (1.00,1.00)	

Abbreviations: HM, Hydromorphone; HM-F, Hydromorhone-Flubiprofen Axetil; SF, Sufentanil; SF-F, Sufentanil-Flurbiprofen Axetil; GYN, gynecologic; VATS, Video-assisted thoracoscopic surgery. Significant results with *p* < 0.05 are in bold.

^*^The odd ratios are for each 1-year increase in age, each 1mg increase in m_H_ or m_F_ and 1μg increase in m_S_ respectively.

### Subgroup analysis

**FIGURE 2:**
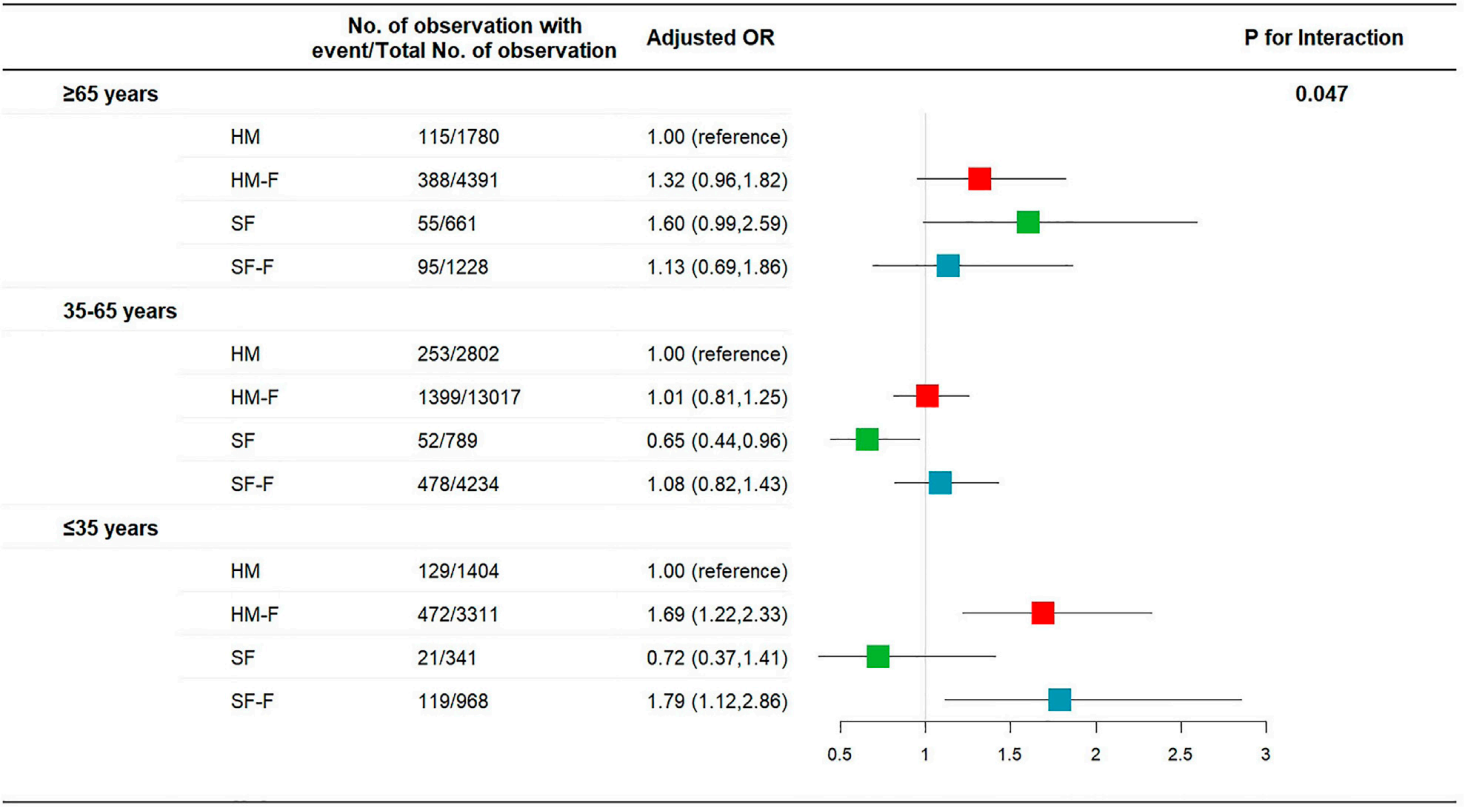
Risk of PONV for patients receiving hydromorphone plus FBP, sufentanil, or sufentanil plus FBP for IV-PCA, as compared to patients receiving hydromorphone, stratified according to patient age.

**FIGURE 3:**
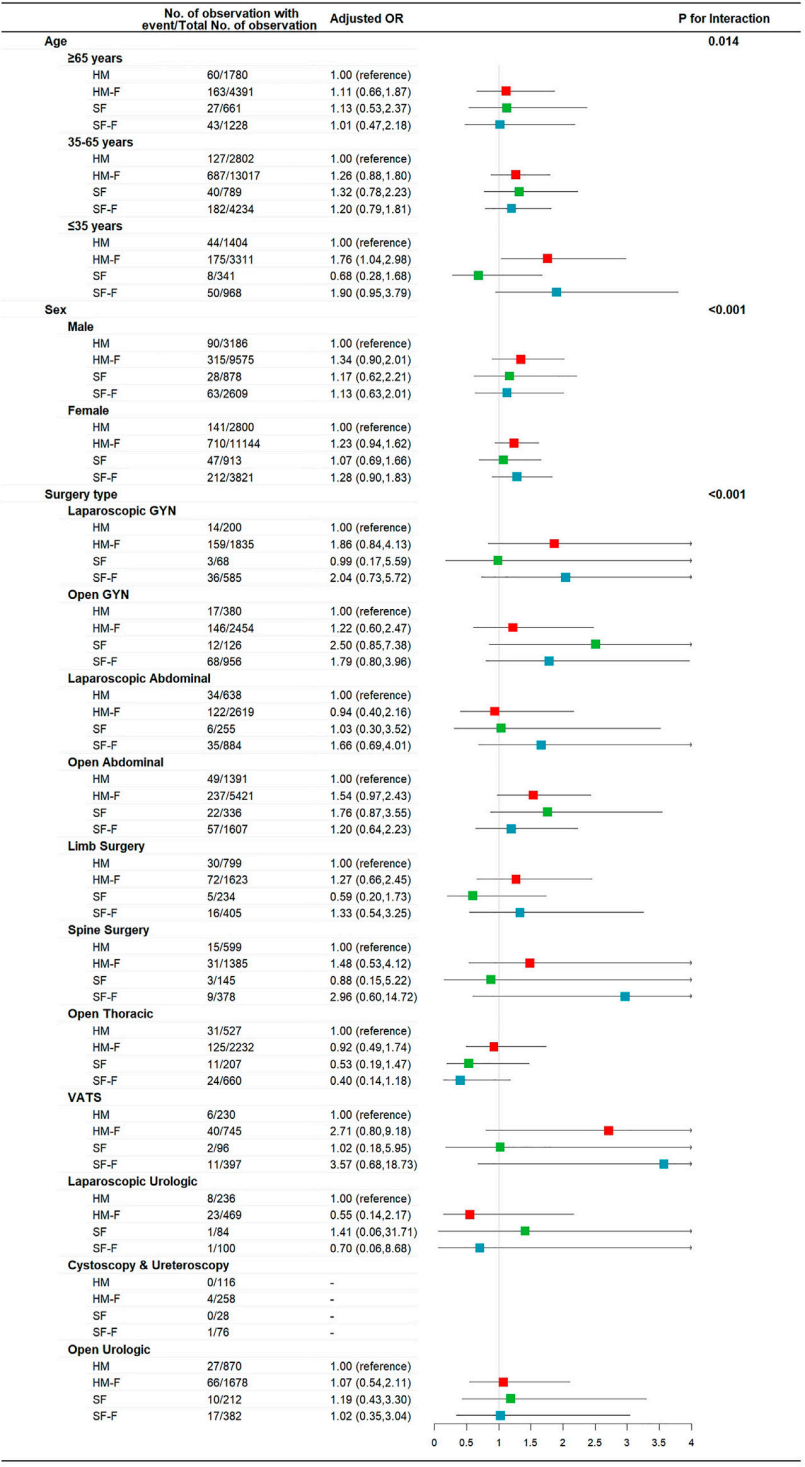
Risk of dizziness for patients receiving hydromorphone plus FBP, sufentanil, or sufentanil plus FBP for IV-PCA, as compared to patients receiving hydromorphone, stratified according to patient age, sex, and surgery type.

The incidence rates of AIS and AIM were not affected by age, sex, or surgery type (*p* for interaction >0.05, Supplementary Figures S1, S2). The incidence of PONV was not affected by sex or surgery type (*p* for interaction >0.05, Supplementary Figure S3) but was significantly affected by age (*p* for interaction = 0.047, Figure 2). The HM-F and SF-F groups showed increased risks of PONV only in the ≤35 years subgroup (OR 1.69 [1.22, 2.33] *p* = 0.001 and 1.79 [1.12, 2.86], *p* = 0.015, Figure 2). Dizziness was affected by age, with the HM-F group showing a higher risk of dizziness in the ≤35 years subgroup (OR 1.76 [1.04, 2.98], *p* = 0.04, and *p* for interaction = 0.014, Figure 3). Meanwhile, the interaction effects between treatment group and sex and between treatment group and surgery type were significant considering dizziness (*p* for interaction <0.001). However, no difference in dizziness between regimens was observed when stratified by sex and surgery type (Figure 3).

### Explorative analysis

The estimated incidences of both AIS and AIM differed most significantly in the first 24 h postoperatively. Adding FBP as an adjuvant lowered the risk of analgesic insufficiency in the first 24 h but not afterward (Supplementary Figures S4A,B). However, the estimated incidence rates of PONV and dizziness remained higher in the HM-F and SF-F groups than those in the HM group at 24–48 h postoperatively as well as in the first 24 h (Supplementary Figures S4C,D).

## Discussion

The results of this historical cohort study of 12,674 patients receiving postoperative IV-PCA demonstrated that adding FBP as an adjuvant significantly improved the analgesic effect, although the potential risk of PONV was increased in patients <35 years of age, and hydromorphone was associated with a lower risk of analgesic insufficiency compared to sufentanil.

Comparison between opioids is difficult. Morphine remains the gold standard in the treatment of acute postoperative pain even though hydromorphone is considered superior in both potency and pharmacokinetics (Hong et al., 2008). Previous publications seldom reported differences between different opioids due to limitations related to sample size, statistical analysis, and high variation in individual responses (Drewes et al., 2013). Most PCA studies utilized small sample sizes, ranging from 500 patients or more, with varied surgical types, making it difficult to compare and pool even in systematic reviews (Dinges et al., 2019). Additionally, PCA used to assess analgesic effectiveness is based on an assumption that the analgesic demand directly reflected pain intensity; thus, both opioid consumption and pain score were important outcome measures in PCA studies (Sechzer, 1990). However, several factors affect the relationship between pain intensity and opioid consumption (Kissin, 2009). First, not all patients in clinical practice achieve complete and pain spontaneous relief due to a fear of opioid-related side effects. As patients were educated to maintain a VAS at rest of ≤3, it is hard to say that two mean VAS <3 had significant differences that were attributable to treatment effects or individual factors. Moore et al. first expressed skepticism regarding the validity of VAS as an outcome measure in 2005 (Moore et al., 2005). A series of studies showed that pain intensity and pain relief were highly skewed data that cannot be appropriately reported as means (Moore et al., 2010; Moore et al., 2011). This was confirmed in the 10-year experience of acute pain service in Italy, in which the mean VAS was relatively low in clinical practice because most patients achieved satisfactory pain relief (Deni et al., 2019). Therefore, the percentage and pain intensity of patients with VAS >3 were concealed by the mean VAS; these populations were inclined to withdraw from clinical trials due o analgesic failure, which introduced bias. Based on these findings, Moore et al. advocated using no worse than mild pain as an outcome, defined as NRS (0–10) ≤3 or VAS (0–100) ≤30, which was better correlated with other pain-related symptoms such as insomnia and depression and more precisely reflected analgesic requirements in clinical practice (Moore et al., 2013a; Moore et al., 2013b). Similarly defined moderate-to-severe pain has been used as an important parameter in population studies and clinical trials (Bulilete et al., 2019; Melcer et al., 2021). Second, while opioid consumption was compared in MME, the conversion factors were controversial, with manual selection introducing bias (Anderson et al., 2001). Finally, varied individual responses to specific opioids played an important role in conflicting results between different trials (Kent et al., 2010).

This study used a cohort including the largest number of common noncardiac surgeries to date. Some surgery types were not included due to the high risk of bias from unmeasured confounders: 1) patients who underwent neurosurgery, cardiothoracic surgery, and oral and maxillofacial surgery were routinely monitored in the intensive care unit immediately after surgery, where they were possibly exposed to opioid analgesics and sedatives; 2) patients who underwent vascular surgery, burn surgery, and plastic surgery were not included because this population was likely to have diabetic neuropathy and chronic pain; and 3) IV-PCA was rarely used after obstetric surgery, thyroid surgery, breast surgery, and eye, nose, and throat surgery. The remaining five specialties included in this study followed similar perioperative care standards in both institutions. To focus on patients who could not achieve a target pain relief by PCA, we chose analgesic insufficiency (defined as VAS score ≥4) as the main measure of analgesic effectiveness, as advocated in the serial studies by Moore et al. (2011; 2013a; 2013b). A two-level GEE model (individual and hospital) was adapted to flexibly include unbalanced analgesic assessments at various time points for up to h, which allowed the construction of a dynamic scope of the postoperative analgesia with less information loss. This model included the real-time drug consumption of hydromorphone, sufentanil, and FBP covariates to adjust the relationship between pain intensity, adverse effects, and drug consumption.

We compared hydromorphone and sufentanil, which represented two typical opioid classes with different pharmacokinetics that are seldom directly compared (Drewes et al., 2013). Consistent with Yan et al., the results of our primary analysis showed better analgesic effects of hydromorphone, with similar risks of PONV and dizziness (Yan et al., 2018). Yan et al. assessed the pain score at five time points (0, 6, 12, 24, and 48 h postoperatively), reporting that the pain score in the sufentanil group was only higher than that in the hydromorphone group at 6 h, which they attributed to the increasing free fraction of sufentanil over time, as reported by Saari et al. (2014). We included more time points in our study and found that the risk of analgesic insufficiency of the sufentanil group remained higher than that in the hydromorphone group at approximately 72 h postoperatively, which was not fully explained by the plasm protein binding rate. Therefore, we speculated that acute tolerance may also contribute to these findings. Coda et al. suggested the development of acute tolerance in the sufentanil group compared to morphine and hydromorphone in patients with oral mucositis pain following bone transplantation (Coda et al., 1997). Preclinical experiments by Kissin et al. revealed that acute tolerance developed within 8 h and was faster in both sufentanil and alfentanil groups compared to morphine (Kissin et al., 1991). However, evidence in a longer timeframe and in comparison with hydromorphone are lacking.

Preemptive or postoperative FBP has long been recommended in multimodal analgesic regimens for the treatment of postoperative acute pain (Yamashita et al., 2006; Wang et al., 2012; Martinez et al., 2019). However, its use as an adjuvant in IV-PCA was not thoroughly studied (Wick et al., 2017). FBP reportedly enhances the analgesic effect of sufentanil and fentanyl in IV-PCA, while its combination with morphine and hydromorphone is rare (Liu et al., 2011; Geng et al., 2015). The results of our primary analysis showed that FBP combined with either hydromorphone or sufentanil was associated with a better analgesic efficacy compared to the respective opioid-only groups. Subgroup analysis revealed that FBP significantly increased the risk of PONV and that the hydromorphone-FBP combination also increased the risk of dizziness in patients aged ≤35 years, findings that were not previously reported. A bolus dose of FBP was believed to reduce opioid-associated adverse effects including PONV; however, no study had examined its influence on adverse effects in continuous use. Therefore, the clinical significance of our findings is unclear (Martinez et al., 2019). As younger age was an independent risk factor of PONV in our GEE model (Table 2), our finding suggests the need for care in the continuous use of FBP in IV-PCA in patients aged ≤35 years. Further clinical and mechanism research are needed to confirm and fully explain this association.

Our study has several limitations arising from its retrospective design. First, the intelligent analgesic research database did not include potential confounders such as primary diagnosis, comorbidities, preoperative medication, preoperative opioid exposure, and postoperative medication prescribed in the ward. Second, while we reclassified surgery types according to specialties and approaches, heterogeneity in pain intensity still existed among each surgery type. Moreover, this 11-level classification resulted in a wide confidence interval in subgroup analysis stratified by surgery type owing to the inadequate sample sizes in some subgroups. Third, due to the diminishing use of morphine in Chinese tertiary hospitals, we could not compare hydromorphone and sufentanil to morphine, which remains the gold standard (Wang et al., 2021). Fourth, the two medical centers in this study are both located in southern China. Given the high variability in individual responsiveness to certain opioids and the unknown underlying mechanisms, the generalizability of our results requires further assessment.

In conclusion, the results of this study demonstrated the superiority of hydromorphone over sufentanil for IV-PCA in the management of acute postoperative pain. Adding FBP as an adjuvant may improve the analgesic effects of both hydromorphone and sufentanil; however, its use was associated with an increased risk of PONV in patients ≤35 years of age. The combination of hydromorphone and FBP was related to an increased risk of dizziness in the same patient population.

## Data Availability

The raw data supporting the conclusion of this article will be made available by the authors, without undue reservation.

## Supplementary material

SUPPLEMENTARY FIGURE 1 Risk of AIS in patients receiving hydromorphone plus FBP, sufentanil, or sufentanil plus FBP for IV-PCA, as compared to patients receiving hydromorphone, stratified according to patient age, sex, and surgery type.

SUPPLEMENTARY FIGURE 2 Risk of AIM in patients receiving hydromorphone plus FBP, sufentanil, or sufentanil plus FBP for IV-PCA, as compared to patients receiving hydromorphone, stratified according to patient age, sex, and surgery type.

SUPPLEMENTARY FIGURE 3 Risk of PONV in patients receiving hydromorphone plus FBP, sufentanil, or sufentanil plus FBP for IV-PCA, as compared to patients receiving hydromorphone, stratified according to patient sex and surgery type.

SUPPLEMENTARY FIGURE 4 Estimated incidence of the outcomes. Incidence-time curves of **(A)** AIS, **(B)** AIM, **(C)** PONV, and **(D)** dizziness.
