# Supplementary figures and images for: New insight into the analgesic recipe: A cohort study based on smart patient-controlled analgesia pumps records
Source: Front Pharmacol. 2022 Oct 10;13:988070. doi: 10.3389/fphar.2022.988070 (PMC9589502; doi:10.3389/fphar.2022.988070)

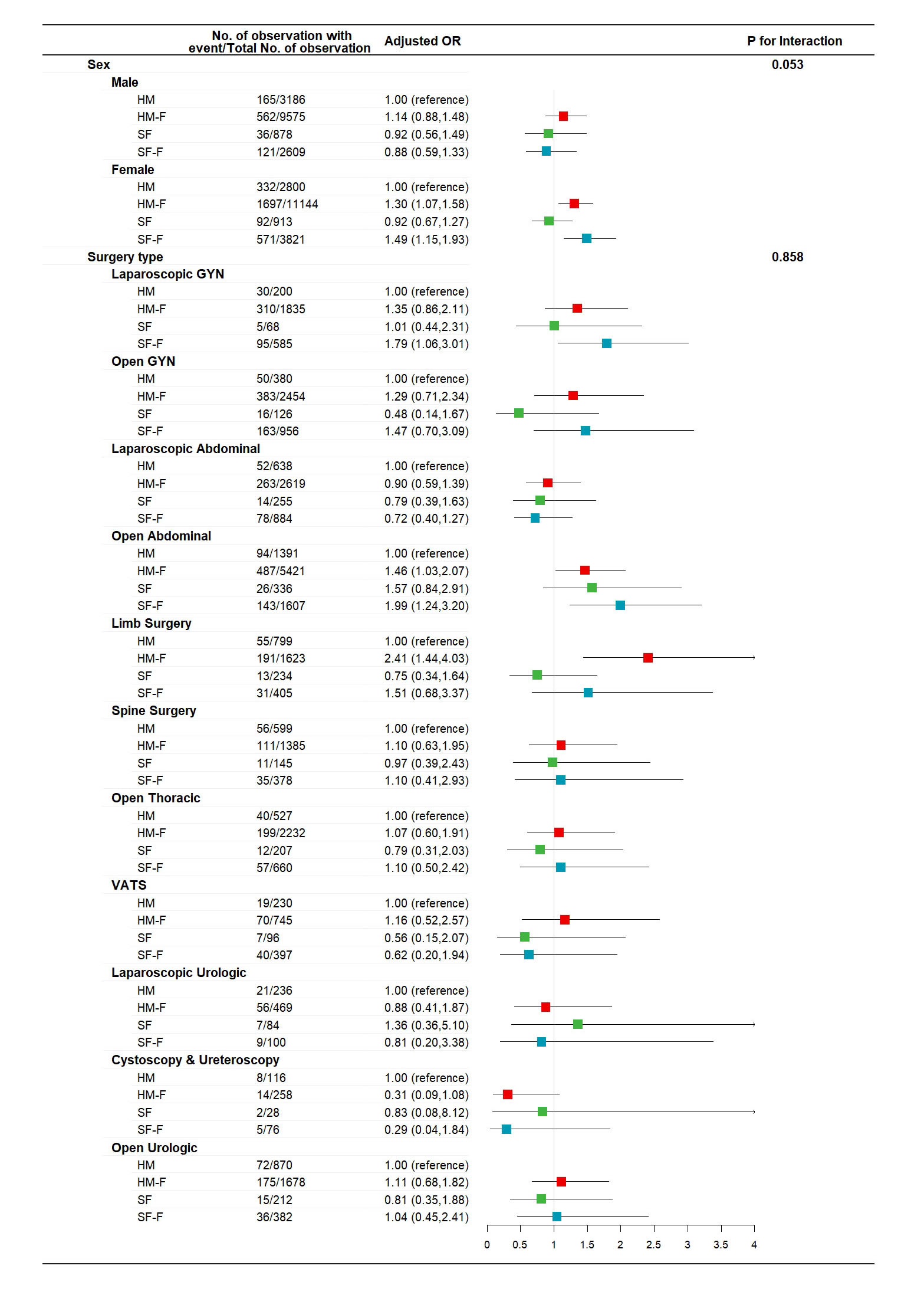

Supplement: Supplementary file 1 [file Image3.JPEG]

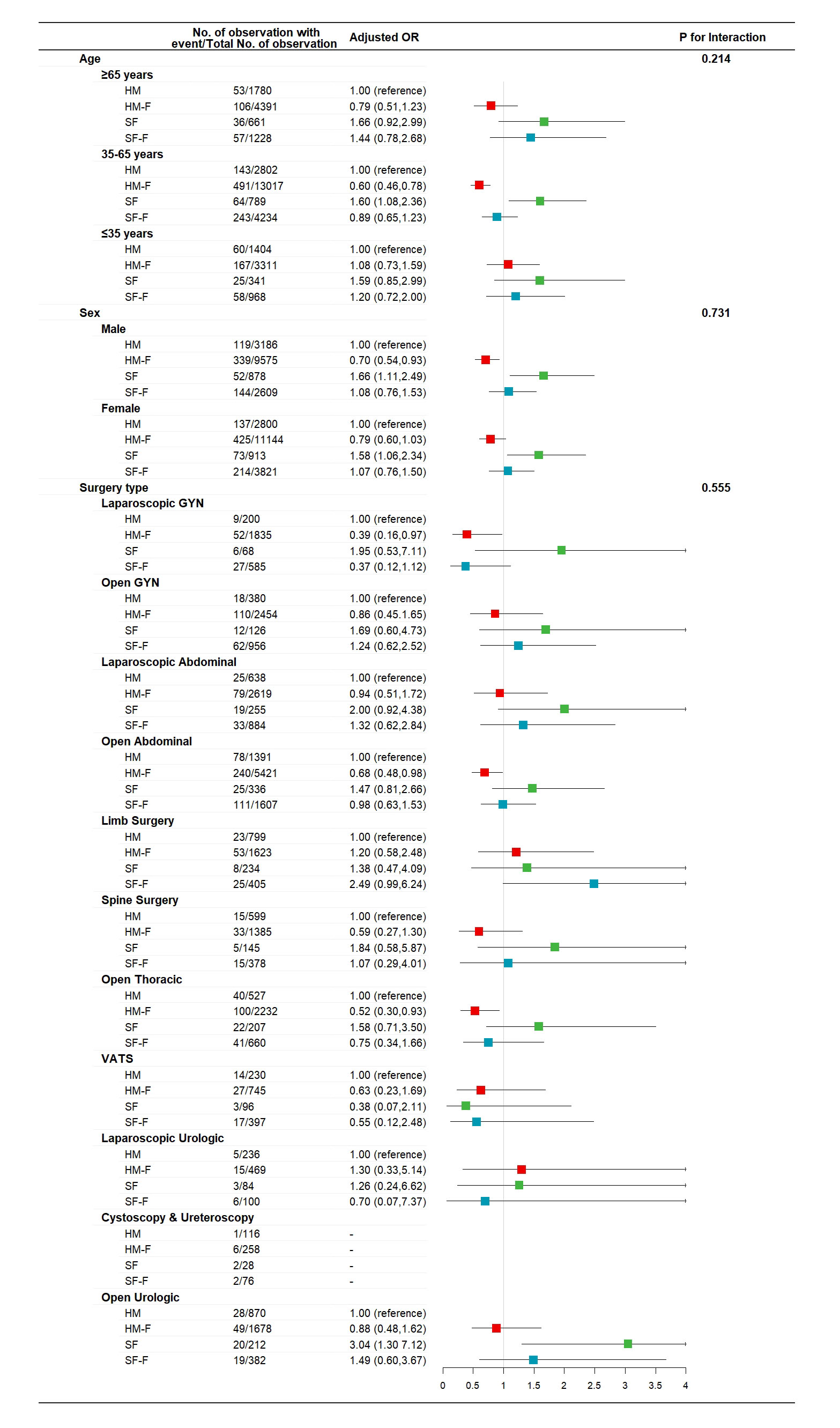

Supplement: Supplementary file 3 [file Image1.JPEG]

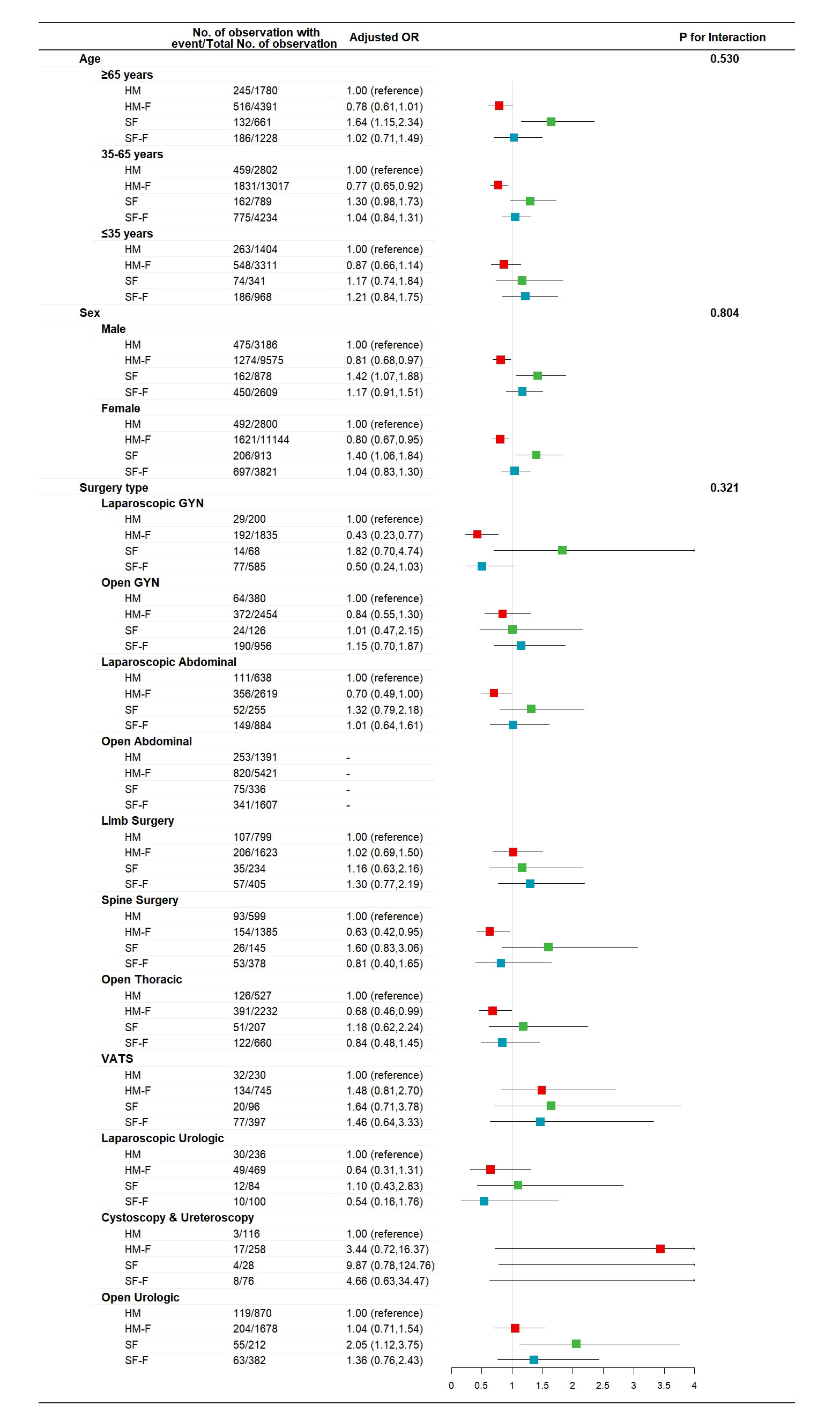

Supplement: Supplementary file 4 [file Image2.JPEG]

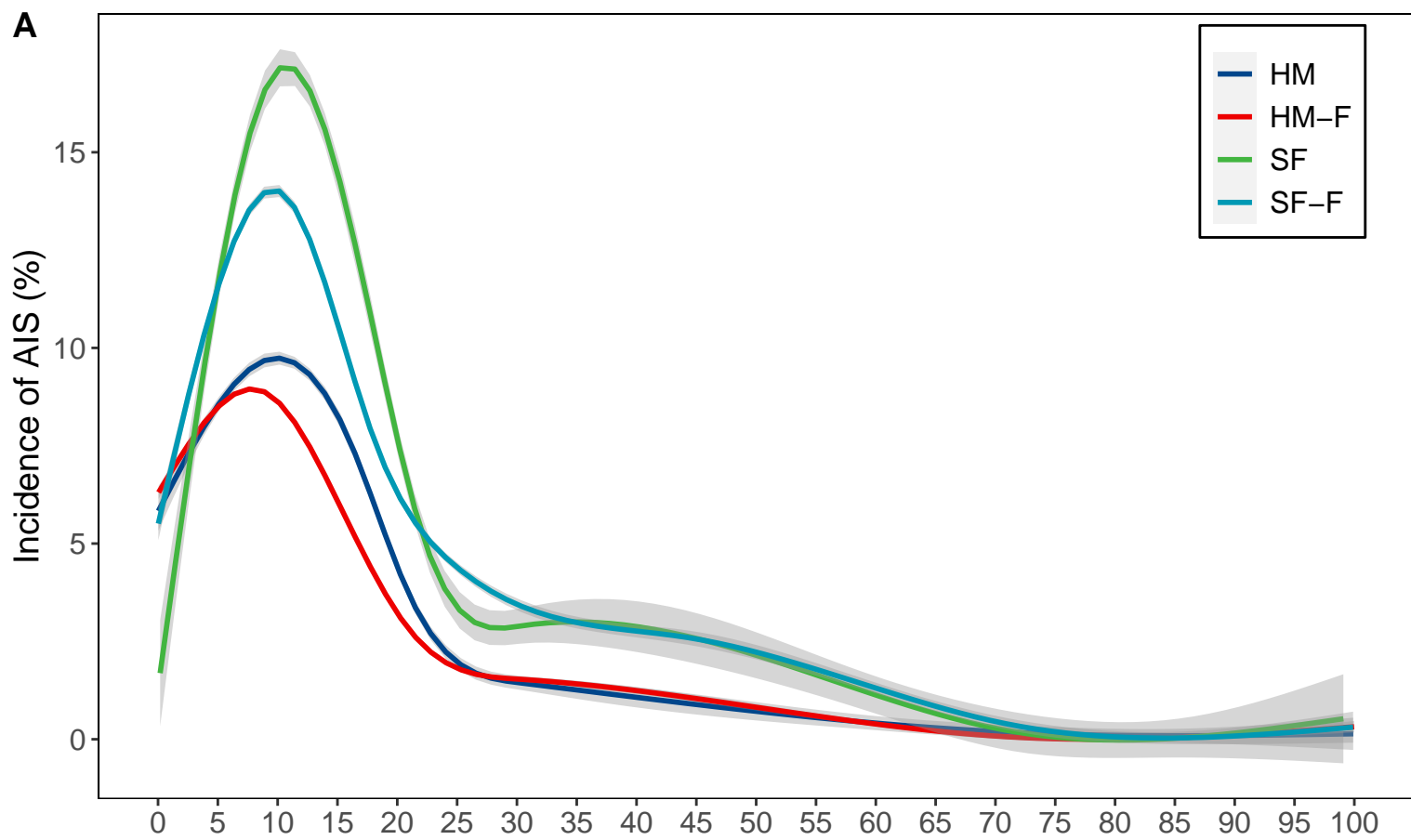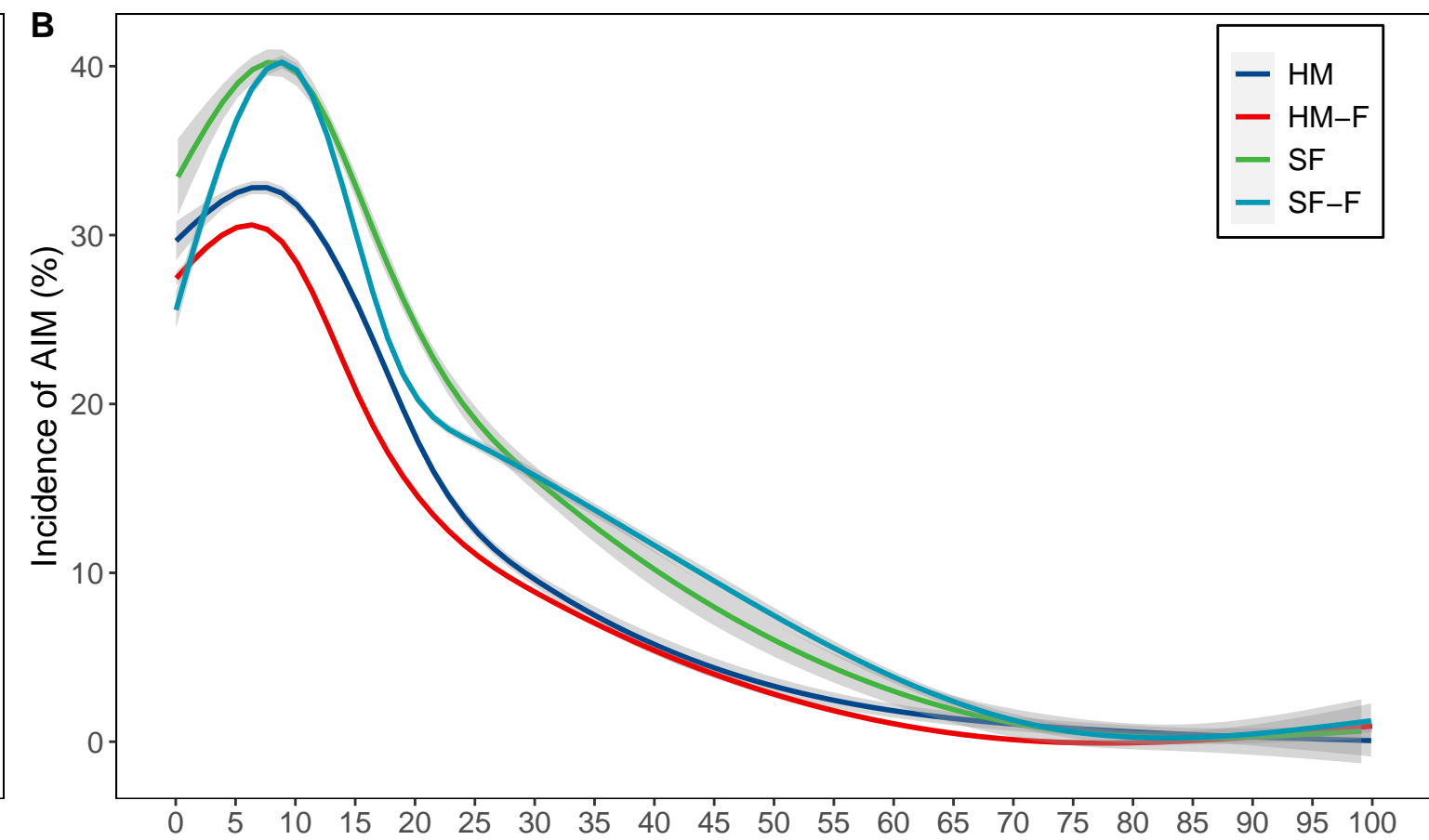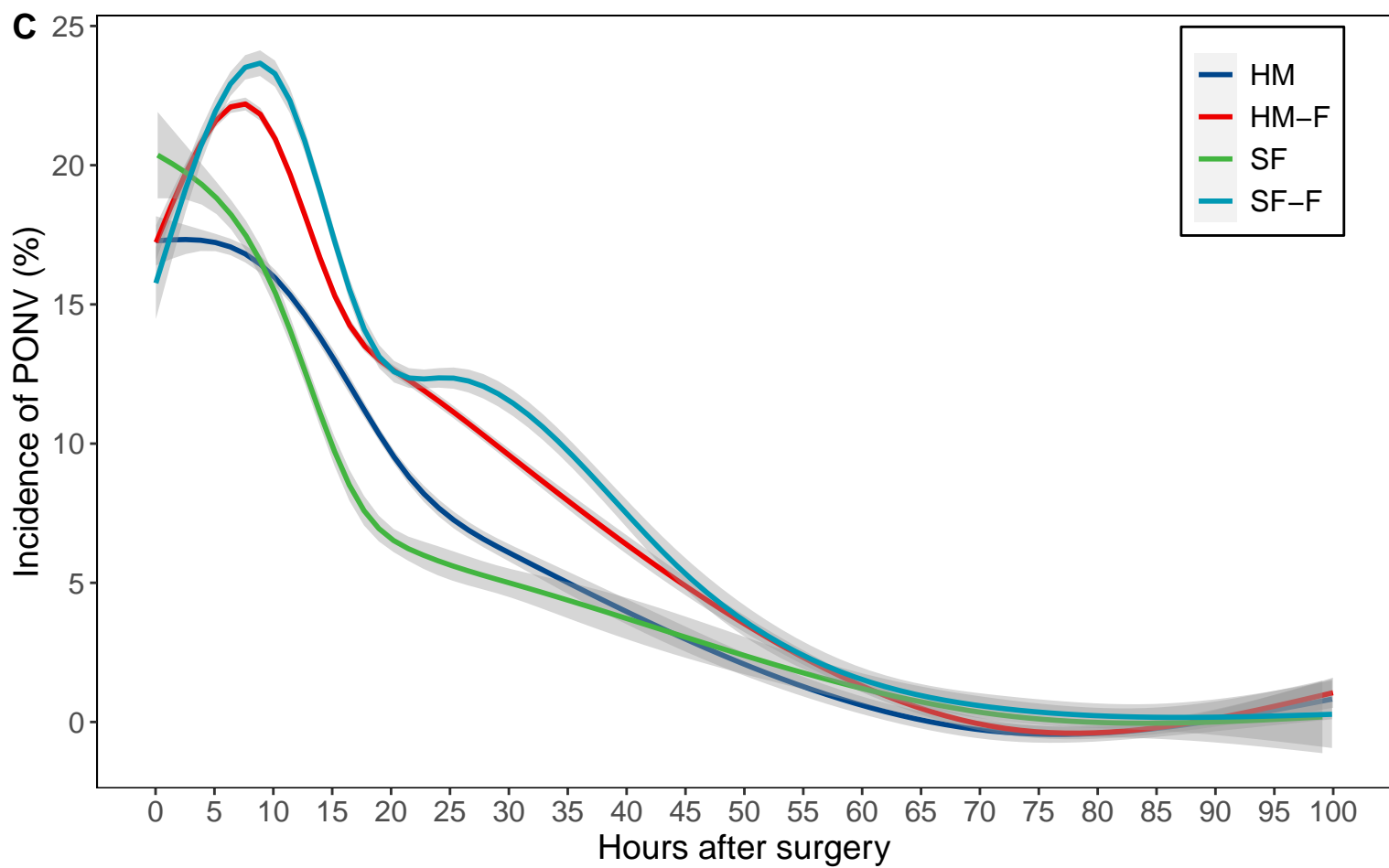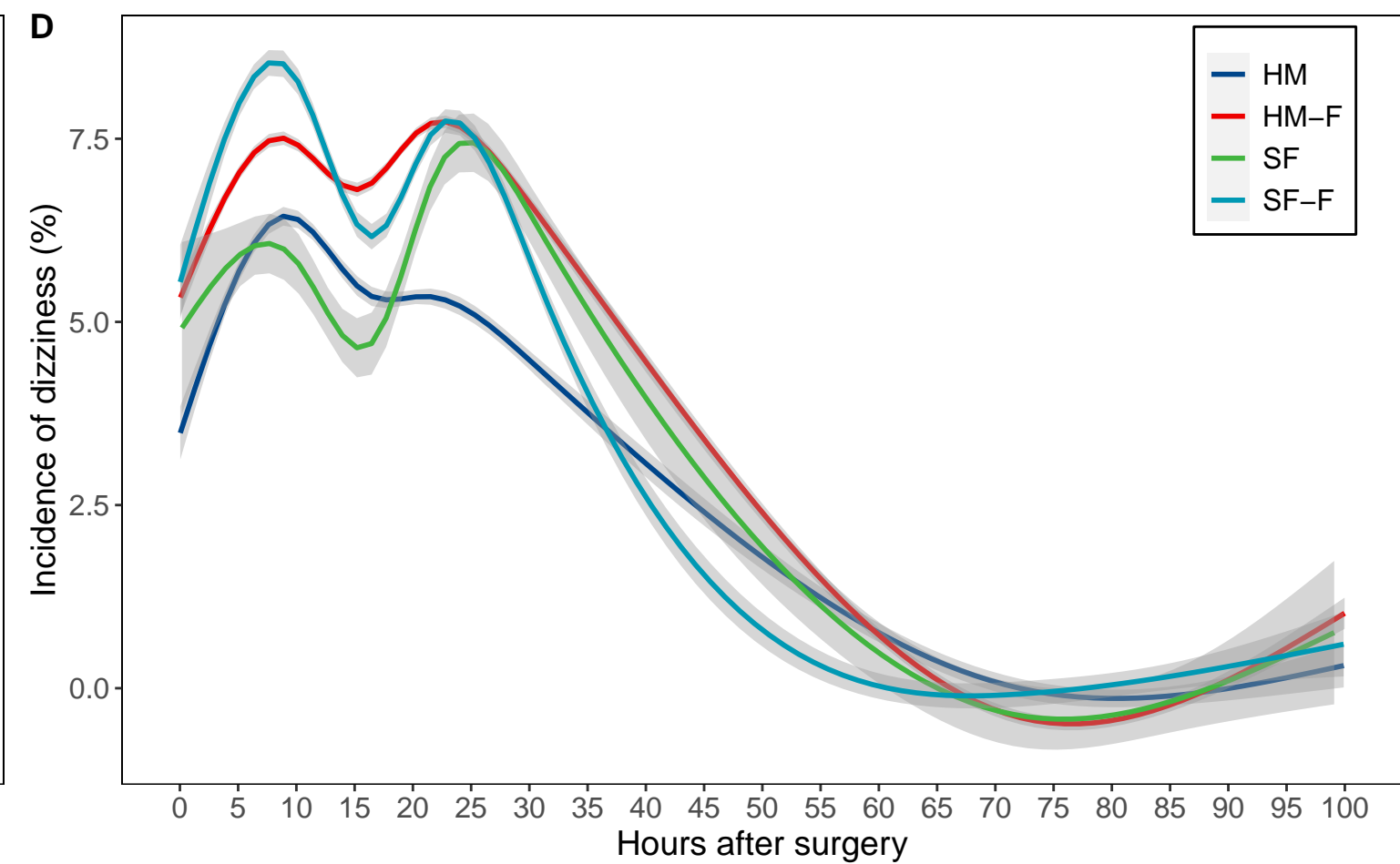

Supplement: Supplementary file 5 [file Image4.pdf]
