# Supplementary material for: New insight into the analgesic recipe: A cohort study based on smart patient-controlled analgesia pumps records
Source: Front Pharmacol. 2022 Oct 10;13:988070. doi: 10.3389/fphar.2022.988070 (PMC9589502; doi:10.3389/fphar.2022.988070)
Supplement: Supplementary file 2 [file Table1.DOCX]

**Supplementary Table1.** Adjusted odds ratio based on the propensity score matched  to the age, gender, height and weight.

|  | Adjusted Odds Ratio (95% CI) | | | |
| --- | --- | --- | --- | --- |
|  | AIS | AIM | PONV | Dizziness |
| HM | 1.00 (reference) | 1.00 (reference) | 1.00 (reference) | 1.00 (reference) |
| HM-F | **0.68 (0.55,0.85)** | **0.81 (0.70,0.94)** | 1.18 (0.96,1.45) | **1.32 (1.01,1.73)** |
| SF | **1.64 (1.17,2.30)** | **1.34 (1.05,1.70)** | 0.96 (0.69,1.33) | 1.19 (0.76,1.89) |
| SF-F | 1.02 (0.76,1.36) | 1.09 (0.90,1.32) | 1.20 (0.93,1.53) | 1.15 (0.83,1.61) |
